# From Lab to Concert Hall: Effects of Live Performance on Neural Entrainment and Engagement

**DOI:** 10.1101/2025.04.03.646931

**Published:** 2025-04-03

**Authors:** Arun Asthagiri, Psyche Loui

## Abstract

Neural entrainment to acoustic rhythms underlies intelligibility in speech as well as sensorimotor responses to music. This property of neural dynamics, where cortical oscillations align in phase and frequency with a periodic stimulus, is well-studied in the context of sensory encoding and perception. However, little is known about how affective components in naturalistic music influence neural entrainment. The present study investigates the effect of live versus recorded music on neural entrainment and tracking using phase-based and linear modeling approaches. 21 participants listened to 2 live and 2 recorded performances of fast and slow movements of solo violin while their EEG data were collected with a mobile system. Participants made behavioral ratings of engagement, spontaneity, pleasure, investment, focus, and distraction after each trial. Live performances were rated as more engaging, pleasurable, and spontaneous than recorded performances. Live trials showed significantly higher acoustic-EEG phase-locking than recorded trials in the frequency range associated with the note-rate of the fast excerpts. Furthermore, the effect of liveness on phase-locking was strongest in individuals who reported the greatest increases in pleasure and engagement for live over recorded trials. Finally, forward linear mapping revealed stronger neural tracking of spectral over amplitude-related acoustic features and a sensitivity to tempo in neural tracking. Altogether, results suggest that experiencing music live strengthens cerebro-acoustic relationships by enhancing rhythmically-driven neural entrainment alongside perceived pleasure and engagement.

**Significance Statement:**

Neural oscillations entrain to rhythms in naturalistic acoustic stimuli, including speech and music. The rhythmic structure of music impacts the timescale of neural entrainment and facilitates the pleasurable urge to move with music, but less is known about how the live experience of music affects neural entrainment. Here, we measure phase-locking and neural tracking between listeners’ EEG activity and naturalistic acoustics during live and recorded solo violin performances, demonstrating that neural-acoustic interactions are driven by musical rhythms and strengthened by the perception of liveness. Together, the study provides insight into neural mechanisms underlying the pleasure of live music, suggesting that the social and affective experience of liveness alters the strength of neural entrainment.

## Introduction

Music is often cited as one of life's greatest pleasures, and despite the popularity of audio and video streaming services, attending live music performances remains one of the most popular activities across age groups and cultures. A growing body of literature shows that liveness (i.e. real-time, in-person experiences) plays an important role in social interaction, mental wellbeing, and collaborative communication (Balters et al., 2023; Stieger et al., 2023). Audiences cite a variety of psychological reasons for being drawn to live performances, including social engagement and novelty seeking (Brown & Knox, 2017). During live performance, audiences synchronize at the neural and physiological levels (Chabin et al., 2022; Czepiel et al., 2021; Tschacher et al., 2023), mirroring social synchrony found in nature (Greenfield et al., 2021; Strogatz, 2015). Entrainment to rhythm acts as a mechanism for naturalistic social bonding (Cirelli et al., 2014; Fujiwara et al., 2020), stimulating joint-action (Rabinowitch, 2023) and a pleasurable desire to move (Witek et al., 2014) that is hypothesized as a core mechanism for music as a means for social bonding (Savage et al., 2021).

Rhythmic entrainment is stronger when listening to music relative to a metronome, suggesting that social and affective aspects of music also factor into entrainment (Stupacher et al., 2017). Behavioral research shows that rhythmic synchrony predicts likeability for human and not machine partners (Launay et al., 2014), and electrophysiological (EEG) studies show that neural entrainment correlates with the pleasurable urge to move only when melodies are human-performed (Cameron et al., 2019). These interactions between social perception and rhythmic entrainment suggest that the social experience of live music may influence neural entrainment to the musical signal. Though live compared to recorded concerts impact the physical energy of audience movement (Swarbrick et al., 2019), it is not known to what extent liveness impacts the rhythmic entrainment of neural oscillations.

Neural entrainment arises from rhythmic acoustic stimulation and is linked to the perception of auditory events (Cabral-Calderin & Henry, 2022; Henry & Obleser, 2012). Canonically, neural entrainment acts as a sensory filtering mechanism mediated by attention (Lakatos et al., 2008, 2019; Schroeder & Lakatos, 2009)--external events reset the phase of neural oscillations and modulate the timescale of neuronal excitation (Obleser & Kayser, 2019). As a result, neural oscillations align in phase and frequency with external stimuli to optimize encoding and prediction (Large & Jones, 1999). In speech and music, the brain responds to acoustic delta-theta modulations (Ding et al., 2017) to enhance encoding and intelligibility (Doelling et al., 2014; Luo & Poeppel, 2007; Vanden Bosch Der Nederlanden et al., 2020). The dynamic neural responses to acoustic stimuli (or cerebro-acoustic relationships) have been investigated with phase-based (phase-locking) and linear modeling (TRFs, see Crosse et al., 2016) approaches. Phase-locking in music is driven by rhythmic frequencies (Tichko et al., 2022a; Woods et al., 2024) and is sensitive to spectral complexity (Wollman et al., 2020) and musical experience (Harding et al., 2019). Phase-locking to rhythm engages the sensorimotor system and contributes to the pleasurable urge to move to music (Zalta et al., 2024), whereas linear modeling has revealed neural tracking of phonemes in speech (Di Liberto et al., 2015), and spectral flux (Weineck et al., 2022) and melodic and rhythmic expectations in music (Bianco et al., 2024).

In the present study, we hypothesized that live music (viz., music that happens in real-time, where musicians or singers perform for an in-person audience, rather than being recorded or pre-recorded) would enhance the strength of rhythmically-driven cerebro-acoustic interactions. Recent research has revealed how audience-performer interactions increase activity in limbic networks and strengthen brain-music correlations in a real-time neurofeedback setting (Trost et al., 2024), but it is not known whether the mere context of the live performer alters oscillatory dynamics in a naturalistic performance setting. We use EEG to test how neural entrainment differs when listening to live and recorded performances in a concert hall.

## Methods

### Participants

21 participants (11 female, mean age 22.9 yrs, range 18-31 yrs) were recruited from online surveys and advertisements in the New England Conservatory of Music community. All participants had a history of formal musical training (n=5, 3-9 yrs; n=16, > 10 yrs) in violin, other strings, winds or voice (n=7, 8, 3, 3 respectively) and at least two years of music theory training. Participants gave informed consent as outlined in IRB #18-12-13, and were paid $25 for their time.

### Stimuli

Participants listened to a total of four excerpts, each a movement from J.S. Bach's Solo Violin Sonatas and Partitas. Two fast movements (approx. 125 beats per minute) and two slow movements (approx. 50 bpm) were selected to match style and structure within tempo conditions as follows: Sonata No. 1 mov. 1—Adagio (slow), Sonata No. 2 mov. 1—Grave (slow), Partita No. 1 mov. 1—Preludio (fast), and Sonata No. 3 mov. 4—Allegro (fast). Two of these movements, one fast and one slow, were presented in live performance by the renowned violinist Joshua Brown. The other two movements were presented with a dual-speaker system. The order of live and recorded stimuli was alternated within each participant and counterbalanced across participants. The order of tempo was a random factor (using a procedure that took into account the live and recorded order and generated a pseudo-random order given the necessary conditions). As a result, every participant experienced all four excerpts once each, and every participant experienced 4 conditions in a 2 x 2 design: Live-Fast, Live-Slow, Recorded-Fast, and Recorded-Slow.

### Procedure

Participants listened in individual sessions to four excerpts (live/recorded x fast/slow) presented in a concert hall (Pierce Hall) at the New England Conservatory, while EEG was recorded with eyes closed. Live performances were given by concert violinist and soloist, Joshua Brown, who played on a 1679 Guarneri Del Gesu violin. Two recorded excerpts, matched by tempo, were played from recordings made by the same violinist in the same concert hall prior to data collection. Live and recorded excerpts were alternated within-subjects and counterbalanced between-subjects while the musical excerpt itself was pseudo-randomized under tempo constraints (see Stimuli).

#### Data Acquisition

Participants rated the listening experience of each excerpt (~ 4 minutes) directly after presentation, reporting affective engagement, enjoyment, familiarity, pleasure, focus, investment, distraction (reverse-scored) and spontaneity. Before the experiment, participants were asked a series of questions assessing musical reward (eBMRQ, Mas-Herrero et al., 2014), musical sophistication and engagement (Goldsmith MSI, Müllensiefen et al., 2014), synesthesia (Synesthesia Battery), and ADHD (ASRS), and gave informed consent in accordance with the Music Imaging and Neural Dynamic Lab's IRB approval (#18-12-13). The experiment generally lasted 1 hour for each participant including EEG setup, questionnaires, and listening.

EEG data were recorded using a dry Quick-32r headset at 500 Hz and collected with the CGX custom acquisition software. Electrode impedances were generally kept under 300 kOhms with some inter-subject variability. A CGX Wireless Stim Trigger device was used to align audio recordings with EEG data at the start of each trial.

All live and recorded performances were recorded for analysis using Pierce Hall's DPA 4011C stereo microphone system. For two excerpts, backup recordings from a separate Blue Yeti microphone were used to due errors with the recording system.

A Yamaha 600S dual-speaker system was used for recorded excerpt playback for the first 11 participants, and a JBL Eon 500 was used as a comparable large dual-speaker PA system for the remaining 10 participants. Levels were matched in decibels between speaker and violinist prior to data collection with ongoing adjustments per trial to minimize differences in amplitude between live and recorded performances. Additionally, the location of the playback system and performer were defined relative to the participant as 14 feet from the audio source (measuring speaker to ear). The bottom of the speakers was situated 4.5 ft above the ground to match the height of the violin during live trials. Finally, the dual speakers were placed close together (approx. 1 ft 10 inches apart) to simulate the single-point sound source of the violin.

### Analysis Plan

#### Behavioral data analysis

Participants gave 5-point Likert ratings after each musical excerpt on pleasure, enjoyment, engagement, familiarity, focus, investment, spontaneity, and distraction (distraction was reversescored). Principal component analysis (PCA) was conducted to find shared sources of variance across questionnaire items. All questionnaire items loaded positively onto the first principal component with weakest contributions from distraction and focus. This first principal component was thus used as a joint "pleasure-engagement" score for following analyses. A 2-factor ANOVA was used to test for effects of tempo and liveness on pleasure-engagement scores. Analyses linking behavior to EEG were performed using a median-split on pleasure-engagement differences between live and recorded performance (within subject) to examine neural correlates of perceived effects of liveness.

### Audio preprocessing

Recordings from each trial were normalized for loudness using Matlab's Audio Toolbox (v. 24.1) to obtain a final gain of -20 LUFS. Stereo recordings were converted into mono tracks by averaging across channels for subsequent analyses.

***Amplitude Envelope:***

To approximate the natural filtering of the cochlea, logarithmic frequency bands were extracted from the raw audio using a gammatone filterbank with 10 logarithmically spaced channels from the MIR Toolbox (v. 1.8.2, Lartillot et al., 2008). Then, the Hilbert transform was applied within each frequency band to extract a complex signal. The final amplitude envelope was computed as the sum of the time varying magnitude of the complex signal across all frequency bands. The resulting acoustic signal was downsampled to 500 Hz to match the EEG sampling rate. Peaks in the frequency spectrum of the amplitude envelope were used to identify stimulus-driven rhythmic activity in slow and fast excerpts including harmonics and subharmonics of the note rate and beat rate.

***Spectral Flux:***

Spectral flux tracks with spectro-temporal changes in the signal. Spectral flux was calculated from the normalized audio by measuring the Euclidean distance between successive time frames in the Mel spectrogram using the native Python Librosa Toolbox (v. 0.10.1, McFee et al., 2023).

### EEG preprocessing:

EEG data was trimmed according to the onset of the excerpt and the length of the excerpt. Data was preprocessed using a custom MATLAB script designed around the EEGlab toolbox (Delorme & Makeig, 2004). Channel locations were loaded from the standard BEM 1005 chart. Data was referenced to the right earlobe (A2) and filtered with a high-cutoff of 58 Hz and a low-cutoff of 0.2 Hz. Importantly, the frequency corresponding to the beat rate of the slow excerpts (~50 bpm or .8 Hz) was included within the broadband EEG signal. Noisy channels were identified and rejected on the following conditions: abnormalities in relation to neighboring electrodes — when the signal was correlated at $r<0.5$ from the surrounding channels' random sample consensus (RANSAC) estimate; a flatline duration of more than 0.5 seconds; line noise which reached more than 4 standard deviations from the signal. Artifact Subspace Reconstruction (ASR) was used to extrapolate missing data regions where the variance relative to calibration data was larger than 10 standard deviations (Chang et al., 2018; Tichko et al.,

2022b). After visual inspection of the data to remove additional outlier channels, all rejected channels were interpolated spherically. Finally, signal artifacts linked to extra-brain sources were removed using Independent Component Analysis.

### Neural Tracking and Entrainment Analyses

The effect of naturalistic music listening on the time-varying brain response was operationalized using two approaches: phase-locking and temporal response function. Generally, the forward temporal response function captures the influence of an external stimulus on broadband EEG data, and canonically has been used to measure the encoding of acoustic speech features. In contrast, cerebro-acoustic phase-locking analyses make theoretical assumptions about neural entrainment that pertain directly to neural oscillations–that the frequency and phase of endogenous neural oscillators are influenced by external rhythmic stimuli (Obleser & Kayser, 2019). In phase-locking analyses, the frequency-specific coherence between the audio and brain stimulus is computed, capturing continuous phase-adjustments of neural oscillators in response to acoustic cues.

***Phase-locking:***

The complex-valued signal was computed from both the real-valued cochlear-filtered audio and EEG signals by complex Morlet wavelet convolution. A wavelet of 5 cycles was used, which is consistent with 5-7 cycles used in acoustic entrainment literature to define the time-frequency precision tradeoff (Cohen, 2014; Harding et al., 2019; Tichko et al., 2022a; Wollman et al., 2020).

Element-wise differences between phase-angle time series were calculated from the complex signals at linearly spaced frequencies from 0.2 to 20.2 Hz with a frequency increment of 0.2 Hz. The coherence value was obtained for each frequency bin of interest by computing the magnitude of the mean complex phase difference vector across samples (see equation below). A single phase-locking value was calculated per frequency, per electrode, and per trial.

$$\mathrm{PLV}_f = \frac{1}{n}\left|\sum_{t=1}^{n} e^{i(\theta_{t,f}^{EEG} - \theta_{t,f}^{Audio})}\right|$$ (Lachaux et al., 1999)

A paired-samples t-test comparing live over recorded performances was conducted within the fast excerpts to assess the effect of performance condition on average phase-locking across salient frequencies associated with the note rate. These salient note-rate driven frequencies were determined from the amplitude spectra of the fast excerpts, which displayed a defined peak across 8-9.6 Hz. Phase-locking values were log-transformed for normality and averaged across the three frontocentral electrodes (F3, Fz, F4), which elicited highest coherence and aligned with the literature (Harding et al., 2019; Vanden Bosch Der Nederlanden et al., 2020; Wollman et al., 2020).

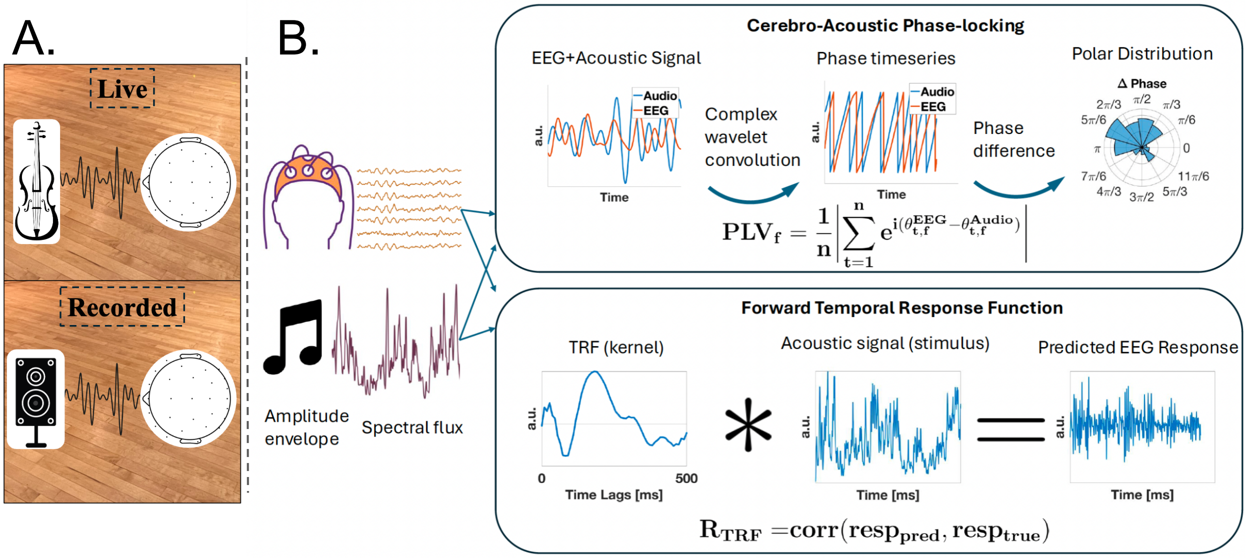

***Figure 1. Methodology–Live and Recorded Trial, Analysis Pipeline.***
*(A) Live and recorded performances were presented separately to 21 participants in a concert hall (eyes closed). EEG was recorded using a mobile system. Live and recorded trials were matched for loudness and acoustic source location. (B) Phase-locking and the temporal response functions revealed naturalistic neural–acoustic relationships. Upper: Phase-locking provided a measure of the phase-angle coherence between acoustic and EEG signals at a given frequency. Lower: Forward temporal response functions were used to model broadband EEG responses to acoustic input. The correlation between predicted and actual neural responses gave an index of the strength of neural encoding.*

***Temporal Response Function:***

The temporal response function was used to examine neural tracking by computing a forward linear model of time-lagged audio features to predict EEG data. Separate models were evaluated by trial and within-subject to compare the strength of tracking under the various conditions using the mTRF toolbox (Crosse et al., 2016). Stimulus audio features (specifically spectral flux and amplitude envelope as described above) and EEG data were both downsampled to 128 Hz and normalized to improve cross-validation (Crosse et al., 2016). A 6-fold cross-validation approach was used to arrive at a ridge regression parameter that reduced overfitting and optimized model performance. The final multi-lag forward TRF model was evaluated on a "test" portion of the data by assessing the pointwise Pearson's correlation between the predicted and true response values.

A two-factor ANOVA was used to compare model correlations when trained with spectral flux versus amplitude envelope acoustic features, across tempi. To compare tracking in the delta and theta ranges, separate models were made for EEG data bandpassed between 0.5-4 Hz for the delta band to include the beat rate of the slow excerpts, and 4-9 Hz for the theta band to include the note rate of the fast excerpts. Two separate two-factor ANOVAs were conducted to test for effects of liveness and tempo at both frequency ranges of EEG data.

## Results

### Pleasure and Engagement are Higher during Live Music Listening

We hypothesized that live music would elicit more positive behavioral ratings than recorded music overall. Indeed, self-reported ratings of live and recorded performances suggested that live music evoked stronger feelings of engagement, enjoyment, pleasure, focus, investment and

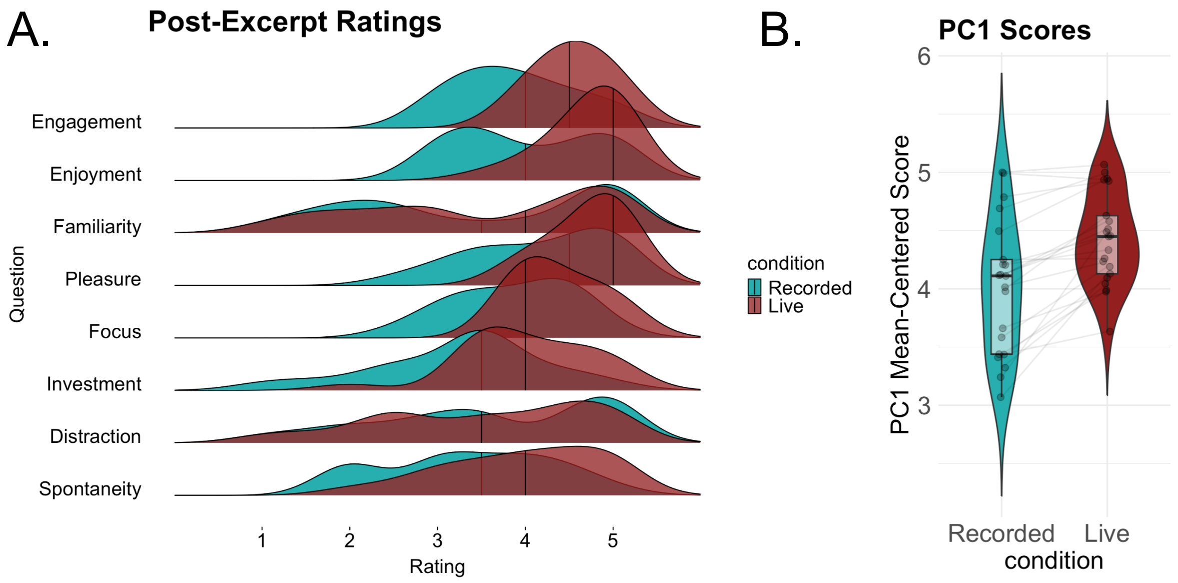

***Figure 2. Behavioral responses to post-excerpt questionnaires.***
*(A) Live compared to recorded trials were rated higher on engagement, enjoyment, pleasure, investment, and spontaneity, and similarly for familiarity and distraction. (B) The first principal component, which summarized a single "pleasure-engagement" dimension, was significantly higher for live over recorded performances (F(1,80)=12.72, p<.001). Error bars show standard error.*

spontaneity (Figure 2A). Ratings of familiarity and distraction were similar between live and recorded performances. After conducting a principal component analysis to examine shared sources of variance in survey responses (see Behavioral data analysis), a two-factor ANOVA was used to assess effects of liveness and tempo on the first principal component scores, referred to as "pleasure-engagement". Live music elicited significantly higher levels of pleasure-engagement than recorded music ($F(1,80)=12.72$, $p<.001$, $\eta_p^2=.137$, Figure 2B) with a non-significant tempo interaction ($F(1,80)=.17$, $p=.680$, $\eta_p^2=.002$), suggesting that regardless of tempo, live over recorded music elicited a more positive overall experience.

### Liveness is Linked to Stronger Phase-Locking at Salient Acoustic Frequencies

Phase-locking value was used as a measure of audio-brain coherence across a range of frequencies (see phase-locking methods). Two distinct peaks in phase-locking were apparent (Figure 3A, bottom), which aligned with peaks in the amplitude spectra of the audio excerpts (Figure 3A, top). The centers of these phase-locking peaks reflects the rhythmic structure of the stimuli: the *beat rate* of the slow excerpts approximated 50 bpm = .83 Hz while the *note rate* of the fast excerpts approximated 4*125 bpm = 8.3 Hz. At a closer look, fast excerpts also elicit strong phase-locking in the delta range, likely reflecting subharmonic groupings of larger

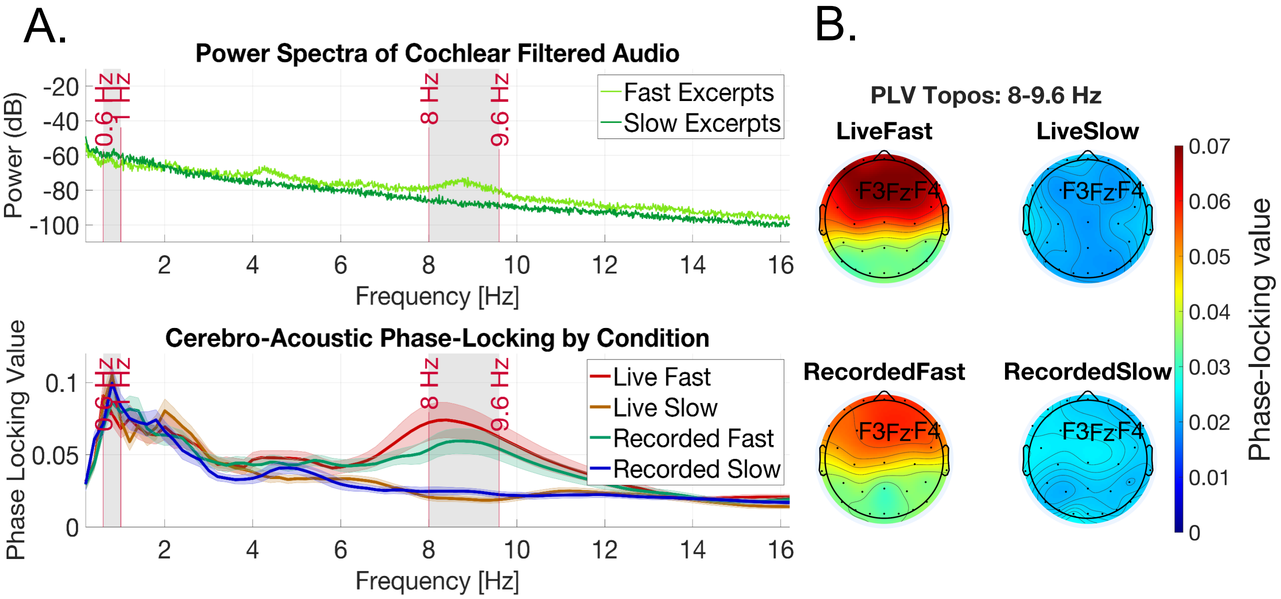

***Figure 3. Cerebro-acoustic phase-locking differs across tempo and liveness.*** *(A) Top: Power spectra of fast and slow excerpts showing tempo-driven differences in frequency-based activity. Bottom: phase-locking values between neural and acoustic activity plotted across a range of frequencies (shaded regions are +-1 standard error). Peaks in PLVs align with delta and theta peaks in the amplitude spectra. Notably, an upper-theta peak in acoustic spectra and phase-locking graphs occurs in the fast excerpts. (B) Plots showing EEG topography of phase-locking values averaged across the 8-9.6Hz peak for each condition. Phase-locking driven by theta rhythms in the fast excerpts was significantly higher during live versus recorded performances (log-transformed).*

structural units (two beats at a time = 1 Hz). Further phase-locking analyses focus on the 8-9.6 Hz frequency range linked to the note-rate of fast excerpts.

After identifying the frequency range that corresponded to the note-rate of the fast excerpts, we used a paired t-test to compare log-transformed phase-locking values averaged over this frequency range and across frontal-central electrodes between live-fast and recorded-fast conditions. Live music resulted in significantly higher phase-locking than recorded music (*t(20)=2.20, p=.040, Cohen's d=.479*).

### Perceptual Differences are Linked to Live Music's Effect on Entrainment

Given the simultaneous observations of brain differences in phase-locking and behavioral differences in pleasure-engagement between live and recorded performances, we additionally tested whether the same individual differences might underlie sensitivity to liveness in both brain and behavior. We performed a median split on the sample by sensitivity to liveness–the difference in pleasure-engagement ratings between live and recorded performances. The effect of live performance was assessed again by paired t-tests comparing log-transformed phase-locking values between live and recorded performances in both high and low liveness-sensitivity groups. The effect was significant for the high liveness sensitivity group (*t(9)=2.853, p < .019, Cohen's d=.902*) but not the low sensitivity group (*t(9)=.926, p=.379, Cohen's d=.293*), indicating that the perceived engagement of live music, over and above that of recorded music, was related to the strength of neural entrainment during listening.

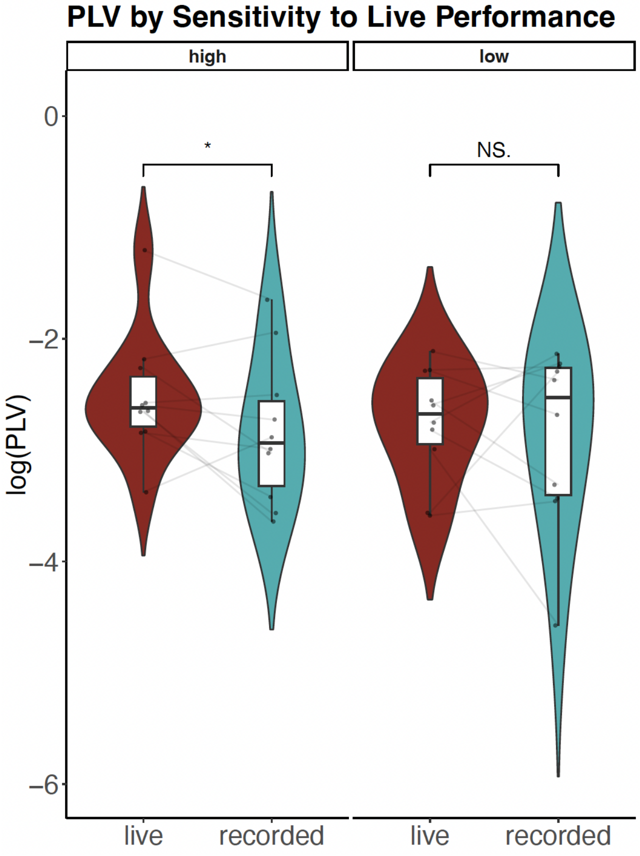

***Figure 4. Phase-locking differences between live and recorded music, split by pleasure-engagement ratings.***
*We conducted an exploratory analysis to test whether participants who perceived the greatest effect of live music exhibited greater differences in phase-locking. A median split of participants by high versus low engagement-pleasure differences between live and recorded performances revealed that the effect of live music on phase-locking was strongest in the participants that perceived the greatest difference between live and recorded trials.*

### Neural Tracking of Spectral Flux over Amplitude Envelope

Forward model multi-lag temporal response functions were used to model neural tracking for both amplitude envelope and spectral flux derived from the audio recordings of every trial

(Figure 5A). First, we trained two models to predict low-frequency broadband EEG data (1-15 Hz) from (1) the spectral flux and (2) the amplitude envelope of the acoustic data. The performance of the two models (Pearson's correlation between true and predicted EEG data) was compared using a two-factor ANOVA to test the interaction between tempo (fast or slow) and acoustic feature (spectral flux or amplitude envelope). Performance medium was not included as a separate factor because we were interested in acoustic influences on neural tracking and live and recorded performances had similar acoustic properties. Spectral flux resulted in significantly higher model correlations than amplitude envelope ($F(1, 164)=11.81, p=.001, \eta_p^2=0.0672$) without a significant tempo interaction ($F(1, 164)=.01, p=.91, \eta_p^2<.001$).

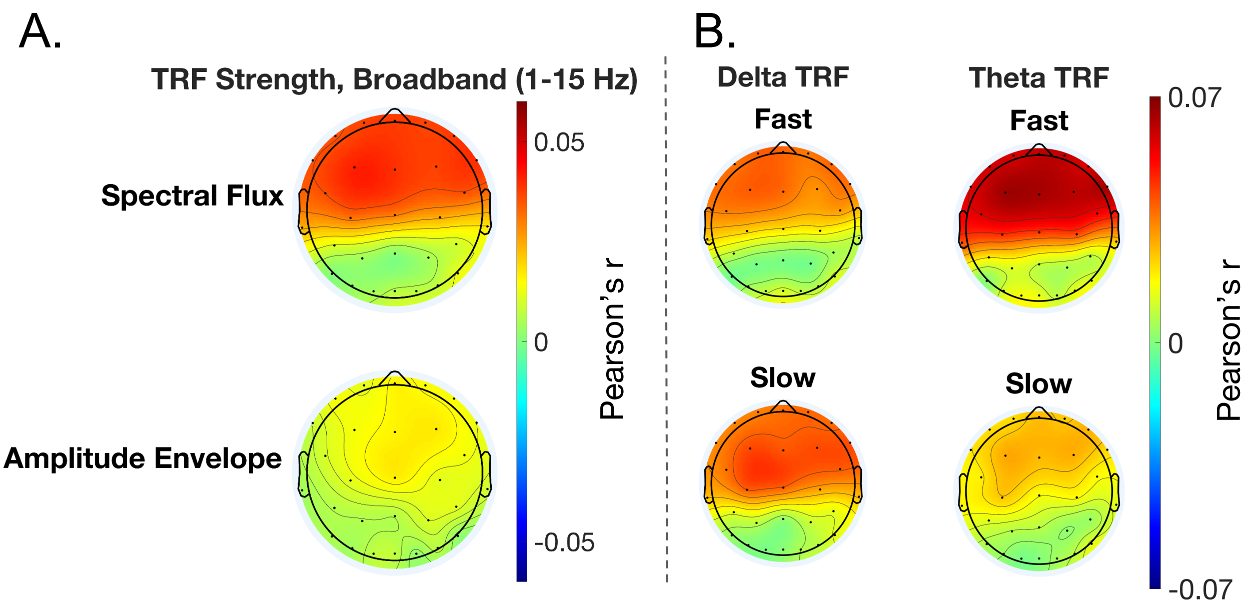

***Figure 5. Forward Temporal Response Function correlations for separate acoustic features and EEG frequency bands.***
*(A) Topography of TRF strength for spectral flux and amplitude envelope features derived from the acoustic signal mapped to broadband EEG (1-15Hz). (B) TRF values for spectral flux mapped separately to delta (.5-4Hz) and theta (4-9Hz) bandpass-filtered EEG signals. Topos reveal difference in delta and theta tracking for slow (50bpm) versus fast (120bpm) excerpts.*

### Frequency of Tracking Differs between Fast and Slow Excerpts

Since peaks in phase-locking reflected neural entrainment at two distinct time scales, we trained separate temporal response function models to predict neural activity from acoustic spectral flux for distinct delta (0.5-4 Hz) and theta (4-9 Hz) EEG components (Figure 5B). The extended frequency ranges included beat-rate activity below 1 Hz and note-rate related activity above 8 Hz (see amplitude envelope fig. 1). Two-factor ANOVAs revealed significant effects of tempo in both delta and theta ranges (delta: $F(1, 80)=24.93, p<.001, \eta_p^2=.238;$ theta: $F(1, 80)=4.61, p=.0349, \eta_p^2=0.055)$, with no significant interactions with performance condition (delta: $F(1, 80)=.61, p=.438, \eta_p^2=0075;$ theta: $F(1,80)=.12=, p=.729, \eta_p^2=.0015)$. Notably, the effect of tempo was opposite for these two frequency ranges: the correlations for the delta TRF models were highest for slow excerpts and correlations for the theta TRF models were highest for fast excerpts.

## Discussion

We provide a novel account of the effects of live music on neural entrainment in a musically-trained population. Participants listened to live and recorded performances of slow and fast excerpts from the solo violin works of J.S. Bach presented in a concert hall by world-renowned violin soloist Joshua Brown. EEG was recorded during music listening, which was used to measure indices of cerebro-acoustic coherence as measured with phase-locking values and temporal response functions. We demonstrate that (1) phase-locking is guided by musical rhythmicity and is stronger during live over recorded performances. (2) Neural entrainment occurred most strongly in the subset of participants who perceived the greatest increases in pleasure-engagement for the live performances more than for their recorded counterparts. (3) Broadband stimulus tracking, measured with the temporal response function, differed significantly between acoustic features (spectral flux and amplitude envelope) and tempo conditions (fast and slow) but not between live and recorded performances. Together, results suggest that neural entrainment increases during live over recorded music as a function of musical rhythmicity, and this increase in neural entrainment accompanies perceived engagement.

### Live music modulates neural entrainment

We measured the phase-locking value between the amplitude envelope of the cochlear-filtered audio and the EEG data over a range of frequencies (.2-20.2 Hz). Phase-locking was strongest at rhythmically salient frequencies in the music for both live and recorded performances (i.e. the note-rate of the fast excerpts and beat rate of the slow excerpts). Low-frequency rhythms in music entrained neural activity, a finding that is consistent with previous research (i.e., Tichko et al., 2022), and here, provides a basis for elucidating differences in how rhythmic properties of the music affect neural dynamics in the phase–frequency domain.

To this end, we tested whether the strength of neural–acoustic phase-locking at rhythmically salient frequencies of the musical excerpts differed between live and recorded performances. Phase-locking was stronger in live compared to recorded performances at the note-rate of the fast excerpts (8-9.6 Hz). The finding suggests that the experience of a live performer significantly alters the strength of acoustic–brain relationships at the timescale of musical rhythms. Neural entrainment, as a low-level auditory encoding mechanism, highlights how the perception of “liveness” may indirectly influence auditory processing of rhythmic frequencies, over and above established acoustic features (i.e., spectral complexity and melodic properties (Vanden Bosch Der Nederlanden et al., 2020; Wollman et al., 2020)), which were controlled in the present design (S1).

Our manipulation emphasized the perceptual difference between live and recorded performance. We reduced the impact of sensory and social factors by having participants listen with eyes closed and without other members in the audience. The highly trained violinist gave consistent performances which reduced acoustic differences, as evidenced by the spectral similarities between live and recorded performances (S1). Thus, we partly recreate the experience of a live concert relative to recorded playback, while controlling for social, acoustic, and visual effects. As a result, our findings point to differences in neural–acoustic relationships that arise from perceptual effects of liveness driven by a single performer–audience relationship rather than by differences in the musical stimulus itself. This complements and extends recent findings from Trost et al., 2024, who focused on neural correlates of live feedback loops, in two ways: first, we show that the presence of a live performer strengthens acoustic–brain coherence without the need for explicit neural feedback, and second, the effect of liveness emerges in the timescale of musical rhythms (here revealed in the theta range), highlighting rhythm as a mediating factor of live performance. This reveals a neural basis for rhythmic entrainment exhibited by audience members in live concerts (Swarbrick et al., 2019).

Differences between live and recorded performances arose in the fast but not slow excerpts. This suggests that live music may have a compounding effect with musical rhythmicity and tempo. The fast and slow stimuli included both tempo-related and stylistic differences. The slow excerpts had more variable rhythms and comprised rubatos, or changes in tempo. Thus, the significant effect of liveness on neural entrainment in fast excerpts may be related to an optimal level of rhythmic complexity for engaging neural entrainment. In that regard, this contextualizes the results from the literature on groove, which also shows that systematic manipulations of rhythmic complexity are linked to the desire to move to music (Benson et al., 2024; Vuust & Witek, 2014).

The increase in neural entrainment during live music has important implications—for one, it supports the notion that rhythmic entrainment is a foundation for live social bonding (Cirelli et al., 2018; Grahn et al., 2021; Savage et al., 2021), consistent with theories of social resonance (Durkheim & Fields, 1995; Wheatley et al., 2012). In addition, the difference in neural entrainment between live and recorded music points to potential differences in the strength of sensory encoding as a function of liveness. Thus, future research may elucidate how liveness supports auditory encoding and rhythmic social signals.

### Perceived experience of live music explains participant-level differences in entrainment

While cerebro-acoustic entrainment has been established as a mechanism for auditory processing and attentional selection (Henry & Obleser, 2012; Lakatos et al., 2008), less is known about how entrainment interacts with the perceived pleasure of an acoustic stimulus such as live music. Given that behavioral results revealed positive changes in affect for the live condition compared to the recorded condition, we looked at whether the variability in the subjective evaluation of live music explained differences in the strength of neural entrainment. Related work has compared neural entrainment to human-performed versus mechanical rhythms, interestingly finding correlations between pleasure and entrainment only in performed but not mechanical music (Cameron et al., 2019). In live settings, perceived pleasure is linked to the strength of inter-subject theta coherence in the audience (Chabin et al., 2022). Our results show that the difference in perception of pleasure and engagement between live and recorded performances is associated with neural entrainment to the acoustic stimulus. Specifically, participants whose pleasure-engagement ratings increased the most from recorded to live performances (grouped by median split) were the ones that exhibited significant differences in phase-locking between live and recorded performances.

The association between individual differences in brain and behavior: that is, the correlation between increased pleasure-engagement ratings and increased phase-locking for live over recorded performance, suggests that neural entrainment may be an indicator of affective experience in music. While neural correlates of musical emotion are typically reported to involve auditory, limbic, and reward systems (e.g., Frühholz et al., 2016; Habibi & Damasio, 2014), the interaction between neural entrainment and affect is less explored. Neural entrainment is considered a low-level mechanism for auditory encoding that gives rise to pleasurable human experiences such as “groove” in music (Zalta et al., 2024). Hence, neural entrainment to music could be one possible route for inducing affect and prosociality (Grahn et al., 2021; J. Trost et al., 2017). However, our study suggests that the relationship between neural entrainment and affect might be also characterized bidirectionally. Liveness alters neural entrainment from top-down perception and results in a markedly different affective response. Conversely, affective responses to live music may induce stronger neural entrainment.

The extent to which neural entrainment and affect follow a top-down or bottom-up relationship has yet to be explored. The manipulation of liveness as a perceptual factor in this study provides some evidence of top-down modulation of neural entrainment through an affective context, analogous to selective-attention as a top-down influence on sensory sampling through entrainment (Calderone et al., 2014; Lakatos et al., 2019). Future research into how affective and reward systems dynamically mediate auditory encoding during naturalistic listening may provide mechanistic insight into the neural dynamics underlying music listening.

### Stimulus tracking is sensitive to acoustics but not liveness

In addition to phase-locking which measured cerebro-acoustic phase alignment at specific frequencies, temporal response functions were used to model broadband neural tracking of acoustic features. First, we replicated recent findings (Weineck et al., 2022) showing that neural activity tracks better with spectral flux than the amplitude envelope for musical stimuli. Then, we used the spectral flux of the audio to separately predict delta and theta EEG activity, revealing that tracking in these two frequency ranges differs depending on the tempo of the excerpt but not liveness. Fast excerpts, which contained more theta activity in their acoustic spectra, had greater tracking in the theta-band than slow excerpts–in contrast, slow excerpts had higher delta tracking. Thus, the neural-tracking differences between delta and theta frequency band demonstrates the importance of the timescale of musical rhythms in driving neural tracking: while neural tracking to acoustic envelopes in speech are similar in in delta, theta and broadband EEG (Di Liberto et al., 2015), music seems to exhibit differences in the frequency of tracking that corresponds to the tempo of the musical excerpt.

A comparison of temporal response function (TRF) and phase-locking results suggests that the TRF is sensitive to differences between structural features in the acoustical signal whereas phase-locking is sensitive to effects at specific rhythmic timescales; this may explain why only the latter was impacted by liveness. Previous work has compared TRFs from different acoustic sources, differentiating phonemes and frequency bands of tracking (Di Liberto et al., 2015; Gwilliams et al., 2022) as well as spectral versus amplitude envelope content (Weineck et al., 2022). Here, we show that spectral features and the tempo of the excerpt affect broadband neural tracking, whereas situational factors like liveness may act at the specific timescale of musical rhythms, which is better revealed through neural phase-locking.

#### Conclusions

Together, the study provides novel insights into the effects of live music on neural entrainment, demonstrating that live over recorded music enhances neural phase-locking to acoustic rhythms in tandem with pleasure and engagement. Results suggest that the neural response to musical rhythmicity strengthens when listening live over recorded music, pointing to a neural mechanism underlying the affective pleasure of live music. Future research may expand towards synchronicity of neural entrainment for diverse musical styles, across multiple listeners, and across multiple performers, and explore the possibility of using the information gleaned from neural entrainment measures to further enhance the experience of live performances.

## Acknowledgements

Thank you to the faculty, Entrepreneurial Musicianship department, recording services and administrative staff at the New England Conservatory for their support and help throughout this project. In addition, I would like to express my gratitude to Joshua Brown for his time and commitment to the project. Joshua Brown’s Guarnerius Del Gesu violin is generously on loan from the Stradivarius Society, Chicago. Additional support from the New England Conservatory’s came through their Entrepreneurial Musicianship Grant award as well as the use of the conservatory’s concert hall space and audio equipment. This work is also supported by NIH R01AG078376, NIH R21AG075232, NSF-BCS 2240330 and NSF-CAREER 1945436, and Sony Faculty Innovation Award to PL.

## Contributions

Designed research: AA, PL
Performed research: AA
Contributed unpublished reagents/analytic tools: AA
Analyzed data: AA, PL
Wrote the paper: AA, PL
